# Peptides for Therapy and Diagnosis of Alzheimer’s Disease

**DOI:** 10.2174/138161212799277752

**Published:** 2012-02

**Authors:** Susanne Aileen Funke, Dieter Willbold

**Affiliations:** 1 Forschungszentrum Jülich, ICS-6, 52425 Jülich, Germany; 2 Institut für Physikalische Biologie, Heinrich-Heine-Universität, 40225 Düsseldorf, Germany

**Keywords:** Alzheimer’s disease, amyloid-β, therapy, diagnosis, peptides.

## Abstract

Alzheimer’s disease (AD) is a progressive neurodegenerative disorder with devastating effects. The greatest risk factor to develop AD is age. Today, only symptomatic therapies are available. Additionally, AD can be diagnosed with certainty only post mortem, whereas the diagnosis “probable AD” can be established earliest when severe clinical symptoms appear. Specific neuropathological changes like neurofibrillary tangles and amyloid plaques define AD. Amyloid plaques are mainly composed of the amyloid-β peptide (Aβ). Several lines of evidence suggest that the progressive concentration and subsequent aggregation and accumulation of Aβ play a fundamental role in the disease progress. Therefore, substances which bind to Aβ and influence aggregation thereof are of great interest. An enormous number of organic substances for therapeutic purposes are described. This review focuses on peptides developed for diagnosis and therapy of AD and discusses the pre- and disadvantages of peptide drugs.

## INTRODUCTION

Alzheimer’s disease (AD) is a devastating neurodegenerative disorder and the most common cause of dementia. The clinical characteristics are difficulties with memory, apathy and depression, impaired judgment, disorientation, confusion and other. The greatest risk factor for AD is age. In 2007, AD affected 27 million people world-wide with steadily increasing tendency. By 2050, the prevalence is estimated to quadruple, thereby raising significant economic problems, not to mention the suffer of each affected individual [1]. 

The pathological hallmarks of AD are the presence of neurofibrillary tangles and amyloid deposits in the brain of the patient, as already defined by Alois Alzheimer in 1907 [2]. Neurofibrillary tangles are aggregates of paired helical filament composed of the abnormally phosphorylated and β-folded tau protein. Tau is a hydrophilic microtubule binding protein which is expressed in six human isoforms of 352 to 441 amino acid residues [3-5].

Aβ is the major component of the amyloid plaques. It consists of 39 to 43 amino acid residues. Aβ, especially Aβ1-42, is prone to aggregation and undergoes formation from monomers to oligomers, larger intermediate forms like protofibrils, and, insoluble fibrils and plaques [6]. Aβ is derived from the amyloid precursor protein (APP) by sequential activities of the β- and γ-secretases [7-9]. As originally suggested by the amyloid cascade hypothesis, it appears likely that Aβ peptides and their aggregated forms initiate cellular events leading to the pathologic effects of AD. According to a previous version of the amyloid cascade hypothesis, fibrillar forms of Aβ, deposited in amyloid plaques, have been thought to be responsible for neuronal dysfunction [7-11]. More recent studies support that diffusable Aβ oligomers including protofibrils, prefibrillar aggregates and so called Aβ-derived diffusible ligands (ADDLs), are the major toxic species during disease development and progression [12-14]. 

Currently, only palliative therapies for AD are available. Acetylcholine inhibitors like Donepezil, Galantamine and the NMDA receptor antagonist Memantine have been approved for clinical use as treatment of cognitive symptoms.

Although it is still controversial if Aβ is the causative agent of AD, inhibition of Aβ production and aggregation are often addressed for therapy development. As a consequence, the majority of AD therapeutic research has been focused on the Aβ peptide. Less effort has been directed towards the development and validation of tau-targeted therapeutic compounds. A number of tau fibril formation inhibitors, derived from multiple chemical classes, have been identified, as reviewed elsewhere [15]. To date, only one tau fibrillization inhibitor, the phenothiazine methylene blue [16], has entered *in vivo* evaluation. Promising first results have been presented at the ICAD meeting in 2008, but up to now have not been published in a peer-reviewed journal. Currently, only one peptide compound addressing tau pathology is known. Davunetide (DAP) is an eight amino acid peptide derived from the activity-dependent neuroprotective protein ADNP. It decreases tau phosphorylation and Aβ levels in tau transgenic mice and 3 x transgenic (tg)-AD mice. The intranasal formulation AL-108 is currently in clinical development [17-21].

Therapy approaches targeting Aβ include reduction of Aβ production by inhibitors or modulators of the β- or γ-secretases, Aβ immunotherapy, and inhibition or modulation of Aβ polymerization [22, 23]. Examples for the latter are scyllo-inositol [24, 25], amino-propane sulfonic acid (Tramiprosate) [26], PBT-1 [27], polyphenol (-) epigallocatechin-3-gallate (EGCG) [28, 29], oligomeric acylated aminopyrazoles [30] and several more.

Peptides, which are specified as (linear) molecules consisting of two or more (<100) amino acids residues, are today reasonable alternatives to chemical pharmaceuticals. They are key regulators of biological functions and offer high biological activity associated with high specificity and low toxicity. The peptide market is growing fast due to an increased number of therapeutic targets, improved delivery methodologies, the establishment of large biological and synthetic peptide libraries, and high throughput screening or selection. Today, 67 therapeutic peptides are on the market, 150 in clinical phases and more than 400 in the pre-clinics. In spite of this progress, the development of peptide drugs can be severely hampered by their short half-life *in vivo*. In general, peptides are rapidly degraded by proteases, and their nature implies problems for administration and delivery, especially to the brain. These problems can at least partially be overcome as peptide chemistry permits a variety of methods for peptide modification or the use of D-enantiomeric amino acid residues [31-33].

A variety of small peptides that inhibit aggregation of Aβ and reduce its toxic effects were already described and a fraction of them shown to be effective in AD rodent animal models. Additionally, Aβ binding peptides, developed for a suitable use in *in vivo* imaging methods and possibly useful for early diagnosis of AD, were described. Both types of peptides, designed for different applications, are reviewed in this article. All peptides discussed in this article and some more are listed in Tables **1** and **2A** to **2F**, but although we scanned the literature exhaustively, this list does not claim to be complete.

## PEPTIDES DEVELOPED FOR AD DIAGNOSIS BY *IN VIVO* IMAGING METHODS (SEE TABLE 1)

Today, the specificity of AD diagnosis can already be improved using glucose metabolism sensing positron emission tomography (PET) experiments [34] and perfusion single photon emission computed tomography (SPECT) [35]. The appearance of amyloid plaques probably occurs many years before cognitive symptoms appear [36, 37]. Therefore, *in vivo* detection and quantification of amyloid species in the brains of patients during the course of the disease, for early diagnosis and the evaluation of the effects of AD-therapies, is an emerging field in AD research. The best characterization of amyloid plaque load in the brain can be expected from imaging approaches using amyloid ligands as contrast agents. To date, many Aβ binding contrast substances failed due to intolerable unspecific binding or poor distribution in the brains of animals. Only a few PET ligands have been applied to clinical studies (for a review, see ref. [38]). The most prominent and best studied is Pittsburgh compound B (PIB) [39], a benzothiazole derivative binding fibrillar Aβ. Novel Aβ binding substances, suitable for *in vivo* imaging, are urgently needed. Small Aβ binding peptides with favorable drug properties could easily be coupled to radionucleides or other markers for imaging of amyloid plaques in living AD patients. 

Phage display technologies allow the identification of peptide ligands for a given target molecule out of a huge library of different peptides expressed on the surface of bacteriophages. Presentation of the peptide library on the surface of bacteriophages ("phage display") as a fusion of peptide and a phage coat protein allows the physical link between the presented peptide and the DNA sequence coding for its amino acid sequence. Diversity of the peptides/proteins can be introduced by combinatorial mutagenesis of the fusion gene. Extremely large numbers of different peptides can be constructed, replicated, selected and amplified in a process called "biopanning". In 2003, Kang *et al*. have employed phage display selection to identify two 20-amino acid peptides specifically binding to the amyloid form of Aβ1-40, but not to monomeric Aβ. One of the peptides (amino acid sequence DWGKGGRWRLWPGASGKTEA) could be produced recombinantly in *E. coli* as a fusion protein with thioredoxin, as well as the chemically synthesized version. The recombinant thiopeptide bound Aβ1-40 amyloid with a K_d _of 60 nM, determined by ELISA. Both versions specifically stained amyloid plaques in brain tissue slices of AD patients. The authors discussed the molecules as potential probes for *in vivo* imaging as well as potential carrier molecules to deliver other therapeutic molecules like antioxidants, chelators, and plaque degrading compounds to the desired location of action [40].

To select for an Aβ-binding D-enantiomeric peptide, which specifically binds to fibrillar Aβ species and plaques, aggregated D-enantiomeric Aβ was used as a target in a mirror phage. Mirror phage display allows the use of phage display to identify peptides that consist solely of D-amino acids. D-enatiomeric peptides are highly resistant to proteases, which can dramatically increase serum and saliva half-life. Additionally, D-peptides can be absorbed systemically after oral administration. D-peptide immunogenicity is reported to be reduced in comparison to L-peptides [41-43]. In the selection process, the Aβ1-42 D-enantiomer was used as a target for selection of peptides displayed on the surface of M13 bacteriophages for those that bind best to D-Aβ1-42. For reasons of symmetry, the D-enantiomeric form of the selected 12-mer peptide will also bind to the native L-form of Aβ1-42 [44]. The most representative peptide in the selection procedure, denoted D1, was demonstrated to bind Aβ with an affinity in the submicromolar range. Employing surface plasmon resonance, binding to Aβ oligomers and fibrils, but not to monomers could be demonstrated. D1 stained amyloid plaques in the brain tissue sections derived from AD patients, whereas other, non-Aβ amyloidogenic deposits, were not stained [45, 46]. D1 was also tested for its *in vivo* binding characteristics in APP/PS1 transgenic mice. Upon direct injection into the brain, D1 bound very specifically to Aβ1-42, staining all dense deposits in the brain but not diffuse plaques, which contain mainly of Aβ1-40 and are not AD specific [47]. This demonstrated that D1 might be suitable for further development into a molecular probe to monitor Aβ1-42 plaque load in the living brain. 

In 2008, Larbanoix *et al*. selected Aβ1-42 binding peptides using a random disulfide constrained heptapeptide phage display library. Two clones (see sequences in Table **1**) were enriched. The K_d_-values for the phage clones, on which several peptide copies are displayed, were in the picomolar range. After peptide synthesis including biotinylation, the binding affinities dropped to the micromolar range. Nevertheless, preliminary *in vivo* studies in transgenic mice, in which the functionalized peptides were used as contrast agents, showed high contrast effects in APP_V717I_/PS1_A246E_ transgenic mice, but not in wildtype controls. For the experiments, however, the blood brain barrier (bbb) had to be permeabilized artificially with 25 % mannitol [48]. Very recently, Larbanoix *et al*. designed another linear hexapeptidic phage display library based on the Aβ1-42 amino acid sequence and selected against aggregated Aβ1-42 as a target. Two of 26 selected clones, presenting highest binding affinities to Aβ1-42, were translated to synthetic peptides with biotine label (Pep1: LIAIMA and Pep2: IFALMG, corresponding Aβ fragment IIGLMV_31-36_) and presented lower K_d_ values (still in the micromolar range) as the peptides described in the first article. Pep1 and Pep2 were highly hydrophobic and are expected to pass the bbb very well. The peptides did not show any sign of toxicity in cell culture. The specific interaction of both peptides with amyloid plaques in human brain tissue was demonstrated by immunohistochemistry [49].

## THERAPEUTIC PEPTIDES (SEE TABLES 2A-D)

### Aβ-sequence Derived Peptides (see Table 2A)

In 1996, Tjernberg and coworkers searched for an Aβ ligand to interfere with Aβ-self interaction and polymerization. The strategy was to identify binding sequences within Aβ and, based on their primary structures, to synthesize Aβ derived peptide ligands. The short Aβ fragment KLVFF (Aβ16-20) was identified to bind full length Aβ and to prevent the fibrillization thereof. Alanine substitution experiments revealed that amino acids Lys16, Leu17 and Phe20 were critical for Aβ interference [50]. A molecular modeling study suggested that the association of full length Aβ and the KLVFF peptide lead to the formation of atypical antiparallel β-sheet structures stabilized by Lys16, Leu17 and Phe20 [51]. 

The effects of KLVFF containing or derived synthetic peptides was further approved in a variety of studies, e.g. by the one of Matsunaga *et al*. [52], see Table **2A**. Additionally, it was proposed that conjugates, bearing several copies of the KLVFF sequence or the retro-inverso version thereof, linked to dendrimers or to branched poly(ethylene glycol) moieties, possess superior affinity and efficiency [53, 54]. In 2008, Austen *et al*. designed KLVFF derived compounds to address very early aggregation intermediates of Aβ, i.e. Aβ oligomers. The idea was to add water soluble amino acids residues to KLVFF, thereby generating the peptides OR1 (RGKLVFFGR) and OR2 (RGKLVFFGR-amid). Both peptides inhibited Aβ fibrillogenesis, whereas OR2 additionally inhibited oligomer formation and Aβ toxicity on SY5Y cells, supporting the idea that particularly oligomers are responsible for the cytotoxic effects of Aβ [55]. Unlike OR2, the retro-inverso D-enantiomeric version (RI-OR2) of the peptide was highly resistant to proteolysis and stable in human plasma and brain extracts [56]. Additionally, it was reported that retro-inversions of OR1 and OR2 increase the inhibitory effects of the peptides [57].

In 2004, Fülop *et al*. developed an Aβ aggregation inhibitor based on the Aβ31-34 sequence IIGL, which also plays a fundamental role in Aβ aggregation and cytotoxicity [58-60]. As in the study described above, the strategy was to link a solubilizing amino acid residue to the original sequence RIIGL. In contrast to propionyl-IIGL, another derivative of the same sequence, PIIGL did not self-aggregate and was not toxic to cells in culture. RIIGL inhibited the formation of Aβ fibrils and reduced Aβ cytotoxicity [61].

In 2008, Fradinger *et al*. prepared a series of Aβ C-terminal fragments (Aβx-42; x = 28-39). The authors of the article tested the hypothesis that C-terminal peptides of Aβ should possess high affinity to full length Aβ and might disrupt oligomer formation, as the C-terminus is supposed to be a key region controlling Aβ aggregation. Cell viability assays identified Aβ31-42 and 39-42 to be the most effective inhibitors of Aβ induced cell toxicity. The peptides additionally prevented the disturbance of synaptic activity by Aβ oligomers. To investigate the *in vitro* mechanism of action, dynamic light scattering, photo-induced cross-linking and discrete molecular dynamics were applied and gave clear hints that the peptides inhibit Aβ induced toxicity by stabilizing Aβ in non-toxic oligomers [62].

The inheritance of the apolipoprotein (apo) E4 allele has been identified as a major genetic risk factor for sporadic AD [63]. All Apo isoforms are discovered to act as pathological chaperones and propagate Aβ fibril formation, with apo E4 being the most efficient isoform [64, 65]. Sadowski *et al*. investigated weather blocking of the interaction between apo E4 and Aβ can have therapeutic effects [66]. In earlier studies, Ma *et al*. demonstrated that the synthetic peptide Aβ12-28 can be used as inhibitor of apo E4-Aβ interaction, inhibiting Aβ fibril formation *in vitro* [67]. Sadowski *et al*. modified the Aβ12-28 sequence: substitution of valine at position 18 to proline rendered the peptide non-amyloigogenic and untoxic whereas the affinity of the peptide to apo E4 was not affected. The use of D-amino acids and end-protection increased the serum half-life of the peptide substantially. Aβ12-28P blocked Aβ-apoE4 interaction and reduced Aβ fibrillogenesis and toxicity *in vitro*. The peptide was bbb-permeable and inhibited Aβ deposition in AD transgenic mice [66].

### β-sheet Breaking Peptides (see Tables 2B-2E)

The first β-sheet breaker peptides were reported by Soto *et al*. in 1996 [68]. In general, the term β-sheet breaker describes compounds, containing an Aβ recognition or binding motif which provides specificity, combined with an Aβ oligomer or fibril disrupting motif which can consist of charged amino acids, prolines, methylated amino acids, cholyl-groups and others. By far the most Aβ aggregation inhibiting peptides are β-sheet breaker, demonstrating the effectiveness of these compounds. In most cases, the Aβ recognition domain is based on the Aβ sequence. 

### β-sheet Breaking Peptides Based on the KLVFF Sequence (see Table 2B)

Starting in 1996, Ghanta *et al*. designed Aβ binding hybrid peptides based on the KLVFF-binding sequence in addition to a disruption domain consisting of a chain of charged amino acids like KKKKKK or RRRRRR. Interestingly, some of the hybrid peptides accelerated Aβ aggregation, but reduced Aβ toxicity. Presumably, the peptides speeded up the association of potentially toxic Aβ oligomers into less toxic Aβ fibrils as demonstrated by a range of biochemical and biophysical methods. The ability of the compounds to increase solvent tension was a very strong predictor on the effect on Aβ aggregation. Chains of charged amino acids without Aβ recognition motif, used in the assays as control, did not exert comparable strong effects [69-75]. In a similar study, Sun *et al*. investigated hybrid peptides composed of the critical Aβ binding domain EIVY and solvent disruptive sequences poly E, K or R. The hybrid peptides EIVY-EEEE and EIVY-KKKK enhanced Aβ fibrillization, whereas EIVY-RRRR inhibited Aβ aggregation and altered the morphology of Aβ fibrils to amorphous aggregates. The ability of the hybrid peptides to interfere with Aβ aggregation was also discussed in the context of their abilities to change the surface-tension in solutions [76], in Table **2E**.

In 2003, Gordon *et al*. replaced the amide bonds of Aβ16-20 with ester bonds in an alternating fashion. The ester peptide Aβ16-20e was monomeric under solvation conditions, inhibited Aβ aggregation and disassembled existing fibrils. Aβ16-20e, could, however, build dimers. These data demonstrated that interference with backbone hydrogen bonding is therapeutically attractive [77, 78]. 

Other β-sheet breakers based on the KLVFF sequence are AMY-1 and AMY-2. Both contain alpha, alpha-disubstituted amino acids at alternating positions and arrest Aβ fibril growth. Instead, large globular aggregates are formed [79]. Rangachari *et al*. investigated the α,β-dehydroalanine (ΔAla) containing peptides P1 (KLVF-ΔA-IΔA) and P2 (KF-ΔA-ΔA-ΔA-F). The design of P2 was based on the experience that peptides containing the FxxxxF motif bind to the groove composed of the GxxxG motif (amino acids G33-G37) in Aβ fibrils [80] α,β-dehydro-amino acid residues are known to be strong inducers of specific peptide conformations. Additionally, they increased resistance to proteolysis *in vivo*. Both peptides under investigation inhibited Aβ aggregation [81].

### Proline Based β-sheet Breaker (see Table 2C)

One peptide compound which was extensively investigated *in vitro*, as well as in animal models, was introduced 1996 by Soto *et al* [68]. The authors developed a peptide partially homologous to the central hydrophobic region of Aβ (amino acids 17-21: LVFFA), containing the amino acid proline to prevent the formation of β-sheet structure and to inhibit Aβ amyloid formation. Proline has special characteristics and is a well-known β-sheet blocker [82]. To increase the solubility of the peptide compound, charged amino acids were added to the ends. The so called “inhibitor of fibrillogenesis” (iAβ1: RDLPFFPVPID) did not aggregate itself, inhibited amyloid formation and disassembled existing fibrils *in vitro*. The relative dissociation constant of iAβ1 was determined by fluorescence spectroscopy to be approximately 80 nM. In order to enhance bbb permeability and to reduce generation of immune responses *in vivo*, the peptide was shortened, and the derivatives iAβ3 (seven amino acids) and iAβ5 (five amino acids) were proven to be similar good or even better Aβ aggregation inhibitors *in vitro* than the basic compound, compared e.g. in ThT assays. The D-enantiomeric version of iAβ1 was as effective as the L-version, and more resistant to proteases [68]. In later studies, iAβ5 was shown to inhibit Aβ cytotoxicity in cell culture. It also reduced Aβ fibrillogenesis in a rat brain model of amyloidogenesis. Male Fischer-334 rats were injected with freshly solubilized Aβ1-42 directly into the amygdala and the animals were sacrificed 8 days later. Co-injection of iAβ5 in 20 % molar excess lead to significantly reduced plaque deposition [83]. In addition, it was shown that iAβ5 induced disassembly of fibrils, reduced Aβ induced histopathological changes like neuronal shrinkage and the extent of interleukin-1β positive microglia cells surrounding Aβ deposits [84]. The chronic intraperitoneal administration of the peptide to the rat model described above lead to a significant improvement of spatial learning acquisition in Morris water maze tests and working memory tests. To perform these studies, iAβ5 was end protected by N-terminal acetylation and C-terminal amidylation [85], as the non-protected peptide was unstable in blood and proteolytically degraded very rapidly [86]. In 2002, two different AD transgenic mouse models (double transgenics overexpressing human APP with London mutation V717I and human PS1 with A246E mutation, and single transgenics only overexpressing human APP V717I) were used to demonstrate that intraperitoneal injected, end-protected iAβ5 reduced amyloid plaque formation, neuronal cell death and brain inflammatory processes. Pharmacokinetic studies in mice and rats demonstrated good stability of the end-protected peptide as well as high capability of the peptide to cross the bbb. The mechanism of peptide action remained unclear, but administration of large doses of the peptide did not lead to antibody production in the treatment and evaluation period [86]. 

In 2004, Datki and Coworkers investigated the effects of a LPFFD based peptide, amino acid sequence LPYFD, on the Aβ induced changes on neuroblastoma cells. Neurite degeneration and tau aggregation were significantly decreased. Additionally, Aβ induced cell toxicity was reduced [87]. The pentapeptide LPYFD-amid protected neurons against Aβ toxicity *in vitro* and *in vivo*. Intraperitoneally administered LPYFD-amid crossed the blood brain barrier in rats at least to a certain extent, and protected against synaptotoxic effects of Aβ up to 3.5 h after i.p. injection [88, 89].

### Methylated β-sheet Breaking Peptides (see Table 2D)

Several teams have studied the effects of N-methyl amino acid incorporation into peptides. For the resulting compounds, the term “meptides” was established. The first methylated β-sheet breaker was described by Hughes *et al*. After it has been suggested that Aβ25-25, amino acid sequence GSNKGAIIGLM, resembles a biologically active region of Aβ that forms large β-sheet fibrils and is highly cytotoxic [90-92], the authors used it as a full length model for Aβ1-42 and synthesized six N-methylated derivatives to prove that those could prevent Aβ wildtype aggregation and cytotoxicity. As the derivatives were homologous to Aβ, they were expected to bind the wildtype form and to prevent further addition of Aβ monomers. It was assumed that N-methylation could block hydrogen bonding at the outer edge of the assembling amyloid, disrupting peptide-peptide interactions that promote Aβ fibrillization. As expected, N-methylated peptide variants had significant influence on Aβ aggregation, as investigated using a variety of biophysical methods. Notably N-methyl-Gly-33 was shown to be amenable to inhibit Aβ aggregation and cytotoxicity. The localization of the N-methyl group was very critical as some of the other peptides did not prevent Aβ aggregation, but altered fibril morphology [93, 94].

In 2001, Gordon *et al*. described the synthesis and biochemical characterization of rationally designed “meptides” based on the KLVFF sequence, containing N-methyl amino acids in alternating positions of the sequence. One of the compounds, termed Aβ16-22m (NH_2_-K(Me-L)V(Me-F)F(Me-A)E-CONH_2_) was shown to be highly soluble in aqueous media and monomeric in buffer solution. It inhibited Aβ fibrillization and disassembled preformed Aβ fibrils *in vitro*. Inhibition was sequence specific and dependent of N-methylization. Protease resistance of the methylated peptide was increased in comparison to the unmethylated Aβ16-22 peptides [78]. The Aβ16-20m peptide, a truncated version of the peptide described above, was synthesized in order to eliminate the charged Glu residue, providing the inhibitor with a net positive charge. Aβ16-20m was effective to inhibit Aβ polymerization and to dissassemble preformed fibrils. In addition, it was highly water soluble despite its composition of hydrophobic amino acids. The peptide passed spontaneously model phospholipid bilayers and cell membranes, suggesting promising pharmacological properties [95]. 

In 2004, Cruz *et al*. developed the peptide “inL”, based on the KLVFF Aβ recognition element. The authors added an additional Lys to the N-terminus, in order to increase solubility, and an N-methyl-20F in order to block Aβ aggregation [96]. The peptide inhibited Aβ toxicity in cell culture very efficiently. In 2009, the corresponding D-peptide “inD”, as well as the retro-inverso peptide “inrD”, was investigated. Both D-enantiomeric peptides were more resistant against protease degradation, as expected. The retro-inverso peptide was shown to be a more effective inhibitor of Aβ aggregation than the other two peptide versions [97].

The peptidic inhibitor PPI-1019, also known as Apan, is derived from the D-enantiomeric Cholyl-LVFFA-NH2 (see chapter *other β-sheet breaking peptides*). The cholyl-group was replaced by a methyl-group and the C-terminal residue was changed from D-alanine to D-leucine. PPI-1019 completed phase I and II clinical trials and was found to be safe, well tolerated and amenable to cross bbb. After peptide administration, levels of Aβ1-40 in the CSF increased, which might be discussed as a sign for Aβ clearance out of the brain [98, 99].

In 2006, Kokkoni *et al*. optimized “meptides” based on the KLVFF sequence in a large approach based on five peptide libraries. Peptide length, methylation sites, end-blocking, side chain identity and chirality were varied. The most interesting compound, judged by Aβ fibrillization and cell cytotoxicity inhibition activity, was D-[(chGly)-(Tyr)-(chGly)-(chGly)-(mLeu)]-NH_2_ and rules could be stated to predict peptide performance. Ideal inhibitors should be D-peptides, possess a free N- but an amidated C-terminus and residues one to four should be large, branched hydrophobic side chains. Only one methylated amino acid was essential [100].

### Other β-sheet Breaking Peptides (see Table 2E)

In 1999, Findeis *et al*. started the development of new β-sheet breaking peptides derived from 15-residue Aβ peptides. The anti-Aβ fibrillization activity of the peptides was enhanced by modification of their amino terminus, where different organic reagents were attached. In subsequent libraries, the size of the inhibitors as well as the amino acid sequences were optimized, and finally, the lead compound cholyl-LVFFA-OH, designated PPI-368, was identified. PPI-368, the acyl-D-amino acid analogue PPI-433 and the amide analogue PPI-457 inhibited Aβ polymerization potently. The latter two were stable in monkey CSF for at least 24 h. Unfortunately, hepatic first –pass elimination was reported for all compounds after biodistribution studies were performed [101, 102].

In 2009, Fryman-Marom *et al*. introduced the D-enantiomeric β-sheet breaker NH_2_-D-Trp-Aib-OH, which combined an indole and α-aminobutyric acid (Aib) [103]. Aib has been shown to induce helical conformations and to disrupt β-sheet structures, the Ramachandran plot indicating that Aib even has the potential to be an better β-sheet breaker than proline [104]. NH_2_-D-Trp-Aib-OH specifically interacted with (untoxic) low molecular weight Aβ oligomers and inhibited their growth to larger, celltoxic Aβ forms *in vitro*. The compound was stable, safe, orally bioavailable, and crossed the bbb in the range of 4 to 8%, depending on the route of administration. It reduced the amount of plaques in the brains of AD transgenic mice and improved their cognitive performance [103].

### Peptides Selected in Combinatorial Libraries

Combinatorial peptide libraries offer a powerful technique to select highly specific peptide ligands for different pharmacological interesting targets [44, 105]. 

Already in 1997, Blanchard and colleaques selected so-called “decoy peptides” using a combinatorial library of approximately 43000 individual sequences composed of the D-amino acids Ala, Ile, Val, Ser, Thr and Gly. The 6 residue peptides were chosen by their ability to complex with tagged Aβ25-35 peptide and had β-sheet forming potential, associating with Aβ and blocking aggregation. Some of the selected peptides abolished the calcium influx, caused by aggregated Aβ25-35 or Aβ1-42 in cell culture [106, 107].

Schwarzman and al. selected inhibitors of amyloid formation by screening of a FliTrx random peptide library. 12 residue peptides were displayed on the surface of *E. coli* by fusion to a flaggelar protein. Synthetic Aβ1-42 was used as a target molecule in five rounds of biopanning. Four groups of Aβ binding peptides were isolated (see Table **2F**), two of which were enriched by positively charged amino acids. Most clones were shown to bind monomeric Aβ, but exhibited very low binding to fibrillar Aβ 1-42 [108].

Orner *et al*. identified peptides that bind to Aβ in either monomeric or fibrillar state using phage display approaches with monomeric or fibrillar Aβ as targets, respectively. Two libraries were designed, guided by the group’s previous studies on the KLVFFK_6_ peptide (see above). The first library displayed sequences with the PoPoPoKLVFFPoPoPo motif, where Po indicates a residue with polar side chain. The second library contained sequences with XXXKLpLpArArPoPoPoPo motif, where X is any amino acid, and Lp and Ar indicate residues with lipophilic and aromatic side chains. Peptides with selectivity for monomeric versus fibrillar Aβ could be identified and most of the selected peptides bound to Aβ10-35 with higher affinity than the parent peptide Ac-KLVFFKKKKK-OH. Peptides selected for Aβ monomer binding did not affect aggregation, whereas peptides selected to bind fibrillar Aβ increased the aggregation of Aβ dramatically, altering the morphology of the resulting aggregate. This effect was clearly correlated with affinity of the peptides to the N-terminal part of Aβ [109].

Taddei *et al*. reported on an approach with the aim to inhibit the catalytic production of H_2_O_2_ by Aβ, which is dependent on Aβ’s superoxide dismutase (SOD)-like activity. A phage display procedure with 6- and 15mer peptide libraries was applied to select for peptides that target the active site of human Aβ’s SOD-like activity, in order to prevent its interaction with redox-active metal ions. As the SOD-like activity site is not present in rat Aβ, a counterselection step with rat Aβ was employed in the phage display procedure. Aβ1-40 or Aβ1-42 were used as targets. 25 peptides which bound to Aβ were identified, and two of the three most enriched peptides, named amyloid neutralizing agents (ANA) 1 to 3, were shown to significantly reduce Aβ’s SOD-like activity in cell culture. A 15-mer peptide additionally reduced Aβ toxicity in cell culture and seemed to be comparably potent as the known Aβ metal-mediated redox activity inhibitor Clioquinol [110].

A very sophisticated system was used by Baine *et al*. to select for peptides that inhibit Aβ aggregation in two combinatorially diverse peptide libraries. The goal was to select peptides which bind the two hydrophobic patches of Aβ and block aggregation by highly charged and polar aspartatic acid residues. Aβ1-42 was genetically fused to EGFP. When expressed in *E. coli*, aggregation of Aβ inhibits the correct folding of EGFP and therefore its fluorescent properties. In the selection process, randomized peptides were co-expressed with Aβ-EGFP. Peptides which were resistant to degradation by cellular proteases and inhibited Aβ aggregation permitted EGFP to be folded properly. Colonies with the brightest fluorescence were chosen for further characterization. Three candidate peptides were selected and characterized, being capable to inhibit Aβ aggregation. One of them even disaggregated preformed Aβ fibrils [111].

Recently a highly specific D-enantiomeric ligand for Aβ has been identified using a mirror image phage display approach with a huge randomized 12-mer peptide library (> 1 billion different peptides). The dominant peptide sequence RPRTRLHTHRNR was obtained, referred to as D3. D3 modulated Aβ aggregation and inhibited Aβ toxicity in cell culture. *In vitro* data clearly demonstrated that D3 is able to precipitate toxic Aβ oligomers into large, high-molecular-weight, nontoxic, ThT negative, nonamyloidogenic amorphous aggregates that fail to act as seeds in Aβ fibril formation assays. D3 did not increase the concentration of monomeric Aβ. Computational simulations of an Aβ nonamer in the presence and absence of D3 proved strong interactions between the arginine-rich D3 and negatively charged groups of Aβ, which were expected to compensate the charge on the Aβ surface and reduce solubility and promote the aggregation of Aβ. Moreover, D3 binding also showed effects on the topology of the Aβ oligomers, which induced a large twist and facilitated the formation of nonfibrillar aggregates [112, 113]. Van Groen *et al*. demonstrated the usage of FITC-labelled D3 for both *in vitro* and *in vivo* staining of Aβ-1-42 in the brains of transgenic AD-model mice [47]. Additionally, D3 was proven to have notable bbb permeability in an *in vitro* bbb cell culture model which further demonstrated the therapeutic potential of D3 [114]. Most recently, oral treatments of Aβ transgenic mice with D3 yielded significant cognitive improvement, reduction of plaque load and plaque-related inflammation [112]. In 2010, Müller-Schiffmann *et al*. reported on the D3 hybrid compound JM169, which combined the D-enantiomeric peptide with a β-sheet breaking compound via a linker substance. The authors demonstrated that the hybrid compound was more efficient *in vitro* than the sum of its components and had novel properties [115].

In 2009, Paula-Lima *et al*. used a phage display approach to select peptides binding to the aggregated form of Aβ. One of the identified heptapeptides with the amino acid sequence GNLLTLD (designated GN peptide) was detected to be homologous to the N-terminal domain of mammalian apolipoprotein A-I. Apo A-I, the major protein component of high-density lipoprotein (HDL), has a central role in reverse cholesterol transport [116, 117] and anti-oxidant as well as anti-inflammatory properties [118]. It was shown that purified human apoA-I and Aβ formed complexes. The interaction of apo A-I also rendered the morphology of amyloid aggregates [119]. Likewise in 2009, Handattu *et al*. evaluated the apo A-I mimetic peptide D-4F, synthesized from D-amino acids and co-administered with pravastatin, as a treatment for AD transgenic mice [120]. D-4F was developed based on the presence of lipid-associating amphipathic α-helices in apo A-I and possessed the ability to avidly bind lipids [121, 122]. Most studies of D-4F were focused on its potential role in atherosclerosis management [123, 124]. Several studies suggested that AD may have an inflammatory component similar to atherosclerosis that is associated with very small vessels such as arterioles [125-127]. In the study, groups of male mice were treated with D-4F and pravastatin via the drinking water. In comparison to the controls, the treated group showed significantly increased cognitive behavior, reduced plaque deposition and reduced inflammatory responses [120].

Very recently, Kawasaki *et al*. constructed a random library to obtain peptide inhibitors specific to inhibit formation of soluble 37/48 kDa Aβ1-42 oligomers. The random library was based on the LPFFD sequence: XX-P-XXX, where X means any amino acid. Novel peptides containing arginine residues were enriched while panning for soluble Aβ1-42. Selected ligands with the strongest affinity to Aβ contained three arginine residues and suppressed formation of 37/48 kDa oligomers and kept the monomeric form of Aβ even after 24 hours of incubation [128].

## CONCLUSION

During the past years, several peptide inhibitors of Aβ aggregation have been investigated for their applicability as new therapeutic lead compounds. In conclusion, it must be stated that only very few, iAβ5 [84-86], Aβ12-28P [66], LPYFDa [88, 89], trp-Aib [103], D-4F [120] and D3 [47, 112, 113] were proven to be effective in rodent mouse models. Only one compound, PPI-1019, is tested in clinical trials. Despite the high diversity of peptides, combined with their simplicity, high specificity, low toxicity and high biological activity [31, 33], peptides are susceptible to proteolytic degradation and in general do not circulate for more than a few minutes in blood [129]. Additionally, peptides in general do not cross membranes very well [130]. 

Several strategies were already applied to overcome high protease susceptibility of peptides and to improve bbb permeability. Different chemical modifications, including incorporation of conformationally constrained amino acids, or modifications of the peptide backbone, have been performed. For example, Adessi *et al*. introduced a methyl-group at the nitrogen atom of one amid bond in iAβ5, resulting in a 10-fold higher *in vivo* life-time in comparison to the unmodified iAβ5 [131]. Moreover, end-protection is commonly used to shield peptides against proteolytic degradation as well as to increase bbb permeability [66, 89, 132]. Another promising strategy to improve the peptide stability is the use of D-enantiomeric amino acids which are considered to be rather protease resistant *in vivo* and in addition often less immunogenic than the respective L-peptides [42, 66, 133]. Additionally, D-peptides can be taken up systematically after oral administration [134].

It was assumed for a long time that Aβ deposited in extracellular amyloid fibrils and plaques were the major pathogenic species in AD. However, over the past decade, accumulating evidence suggests that Aβ oligomers are the toxic moiety responsible for synaptic dysfunction and neuronal cell loss [12]. At the moment it is unclear whether peptide inhibitors should be targeted to monomeric Aβ, oligomeric Aβ or Aβ fibrils deposited in plaques. It is also unclear if Aβ1-40 or Aβ1-42 should be addressed and weather compounds need to cross the bbb in order to be effective. More work is necessary to elucidate the nature of the most synaptotoxic Aβ species during AD development and progression. The ongoing research on peptides which target distinct Aβ species, as well as the investigation of their influence on Aβ aggregation and toxicity, will provide further understanding of the molecular mechanisms involved in AD.

## Figures and Tables

**Table 1.** Peptides Selected for *In vivo* Imaging

Name	Sequence	Description	D/L	Results	Reference
none	DWGKGGRWRLWPGASGKTEA and PGRSPFTGKKLFNQEFSQDQ	Selected by phage display	L	Binds amyloid form of Aβ40, labels amyloid plaques in AD brains slices, discussed as carrier protein for plaque treatment and *in vivo* imaging	Kang *et al*., 2003 [40]
D1/ ACI-80	ACI-80: QSHYRHISPAQV	Selected by mirror image phage display	D	Aggregate specific, stains human Aβ plaques selectively, stains plaques in mice ex vivo	Wiesehan *et al*., 2003 [46] Van Groen *et al*., 2009 [47]
none	C-IPLPFYN-C / C-FRHMTEQ-C	Selected by phage display	L	K_d_ for Aβ42 in micromolar range, specific interaction with plaques on brain sections (immunochemistry), encouraging preliminary MRI *in vivo* study in mice after opening of bbb by mannitol	Larbanoix *et al*., 2008 [48]
Pep1 Pep2	LIAIMA IFALMG	Selected by phage display, library based on Aβ sequence	L	K_d_ for Aβ42 in micromolar range, specific interaction with plaques in human brain sections (immunochemistry), inhibit Aβ aggregation	Larbanoix *et al*., 2011 [49]

bbb: blood brain barrier. D/L: describes peptide conformation.

**Table 2A.** Peptides Influencing Aβ Aggregation and Toxicity. Aβ-sequence Derived Peptides

Name	Sequence	Description	D/L	Results	Reference
Aβ(16-20)	**KLVFF**	Based on Aβ sequence	L	Prevents Aβ fibrilization, identification of key amyloidogenic region	Tjernberg *et al*., 1996, 1997 [50, 51]
Aβ- (15-22) Aβ- (16-23) Aβ- (17-24)	Include **KLVFF**	Based on Aβ sequence	L	Inhibit Aβ aggregation *in vitro*	Matsunaga *et al*., 2004 [52]
K4	**KLVFF**-dendrimer	Aβ sequence conjugate	L	Inhibitory effect of compound is potentiated in comparison to monomer	Chafekar *et al*., 2007 [53]
none	**KLFVV ** retro-inverso version linked to branched hexameric PEG	Aβ sequence conjugate	D	Inhibitory effect on Aβ aggregation is potentiated	Zhang *et al*., 2003 [54]
OR1, OR2	RG**KLVFF**GR or RG**KLVFF**GR-NH_2_	Based on Aβ-(16-20) region	L/D	Inhibit Aβ oligomerization, aggregation and toxicity. Retro-inverso-peptide resistant to proteolysis and more active	Austen *et al*., 2008; Matharu *et al*., 2010 [55, 57]; Taylor *et al*., 2010 [56]
Aβ- (31-35)	R**IIGL**	Based on Aβ sequence IIGL	L	Inhibits Aβ aggregation *in vitro*	Fülöp *et al*., 2004 [61]
none	Aβ(x-42) X=28-39	C-terminal Aβ sequence derived peptides	L	Stabilize Aβ in non-toxic oligomers, inhibit Aβ neurotoxicity	Fradinger *et al*., 2008 [62]
Aβ12-28P	Aβ(12-28) V18P mutation, end-protected	Peptides block Aβ-Apo E4 interaction by competing for the binding site	D	Inhibition of aggregation *in vitro*, reduction of plaque formation in tg mice	Sadowski *et al*., 2004 [66]

D/L: describes peptide conformation. PEG: poly (ethylene glycol). Aβ-derived peptide sequences are written in bold. Tg: transgenic

**Table 2B.** Peptides Influencing Aβ Aggregation and Toxicity. β-sheet Breaking Peptides Based on the KLVFF Sequence

Name	Sequence	Description	D/L	Results	Reference
nn	**KLVFF** based, with chain of charged amino acids as disruption motif	β-sheet breaker	L	Enhance fibrillization of Aβ oligomers and therefore reduce Aβ toxicity. Effectiveness of inhibitor is dependent on its surface tension modifying properties	Ghanta *et al*., 1996; Pallitto * et al*., 1999; Lowe *et al*., 2001; Kim *et al*., 2004; Moss *et al*., 2003; Kim *et al*., 2003; Gibson *et al*., 2005 [69-75]
Aβ16-20e	**KLVFF ** with ester sub-stitution	No hydrogen bond can be formed	L	Inhibit Aβ aggregation, disassembles fibrils. Expected to hydrolyze rapidly *in vivo*	Gordon *et al*., 2003 [77, 78]
AMY-1 AMY-2	**KLVFF** based	β-sheet breaker α,α-disubstituted amino acids	L	Inhibition of fibrillization, globular aggregates are formed	Etienne *et al*., 2006 [79]
P1, P2	**KLVF**-ΔA-I-ΔA and KF- ΔA- ΔA- ΔA-F	Disruption of aggregation by different local confirmation	L	Inhibit Aβ aggregation	Rangachari *et al*., 2009 [81]

D/L: describes peptide conformation. ΔAla: α, β-dehydroalanine. Aβ-derived peptide sequences are written in bold. Table

**Table 2C.** Peptides influencing Aβ aggregation and toxicity. β-sheet breaking peptides based on proline

Name	Sequence	Description	D/L	Results	Reference
iAβ5	Ac-LPFFD-amid	Proline β-sheet breaker	L/D	Aβ fibril inhibition and de-fibrillization * in vitro*. Reduction of plaque load and Aβ induced pathological processes in rat model and in AD tg mice. Improvement of rat spatial memory impairments	Soto *et al*., 1996; Soto *et al*., 1998; Poduslo *et al*., 1998; Sigurdsson *et al*., 2000; Permanne *et al*., 2002; Chacon *et al*., 2004 [68, 83-86, 135]
iAβ5	LPFFD-derivatives	Proline β-sheet breaker	L	Methylation of amide nitrogen increased *in vitro* and *in vivo* stability while maintaining iAβ5 activity *in vitro*	Adessi *et al*., 2003 [131]
iAβ_5_-PEG	LPFFD-PEG	Proline β-sheet breaker	L	Biological activity of iAβ_5 _(*in vivo*) is not affected by PEG. Aimed to improve pharmacological properties, especially *in vivo* degradation	Rocha *et al*., 2009 [136]
none	LPYFD	Proline β-sheet breaker	L	Decreased neurite degeneration, tau aggregation and cell viability reduction induced by Aβ	Datki *et al*., 2004 [87]
LPYFDa	LPYFDamid	Proline β-sheet breaker, amidated	L	Protects neurons *in vitro* and *in vivo* after intraperitonial administration to rat models	Szegedi *et al*., 2005; Juhász *et al*., 2009 [88, 89]

D/L: describes peptide conformation. Tg: transgenic. PEG: poly (ethylene glycol).

**Table 2D.** Peptides Influencing Aβ Aggregation and Toxicity. β-sheet Breaking Peptides Based on Methyl-Amino Acids

Name	Sequence	Description	D/L	Results	Reference
none	N-methylated **GSNKGAIIGLM**	First methylated β-sheet breakers	L	Prevent Aβ aggregation *in vitro*, inhibit cell toxicity	Hughes *et al*., 2000 [93, 94]
Aβ16-22m Aβ16-20m	**KLVFF ** based e.g. **K**_Nme_**LV**_NME_**FF**_NMe_AE	Methylated β-sheet breaker, N-methyl groups in alternating positions	L	Prevent Aβ fibril forming, disassemble fibrils *in vitro*	Gordon *et al*., 2001, 2002 [78, 95]
inL (all L) in D (all D) inrD (retro-inverso)	L**KLVFF **based, N-methyl-20F	Methylated β-sheet breaker, single N-methyl-amino acids	L/D	Reduces Aβ toxicity in cell culture	Cruz *et al*., 2004; Grillo-Bosch * et al*., 2009 [96, 97]
PPI-1019 “Apan”	Methyl-LVFFL	Methylated β-sheet breaker	D	Completed phase I and II human clinical trials	Jhee *et al*., 2003 [98, 99]
e.g. SEN304	e.g. D-chGly-D-Tyr-D-chGly-D-chGly-D-mLeu	“meptide”, methylated β-sheet breaker	D	Highly active inhibitors of Aβ aggregation and toxicity	Kokkoni *et al*., 2006 [100]

D/L: describes peptide conformation. chGly: cyclohexylglycine; mLeu: N-methylleucine; NMe: N-methylated amino acids. Aβ-derived peptide sequences are written in bold.

**Table 2E.** Peptides Influencing Aβ Aggregation and Toxicity. Other β-sheet Breaking Peptides

Name	Sequence	Description	D/L	Results	Reference
e.g. PPI-368 PPI-457	e.g. Cholyl-LVFFA	β-sheet breaker Aβ-binding sequences with cholyl-bulky group	L/D	Aβ specific, inhibit Aβ aggregation potently, reduces Aβ cell toxicity	Findeis *et al*., 1999, 2001, 2002 [98, 101, 102]
Aβ-(38-42)	G**VVIA**, R**VVIA**	Based on Aβ sequence, amidated at C-termius	L	Inhibit Aβ aggregation and toxicity	Hetényi *et al*., 2002 [132]
Trp-Aib		β-sheet breaker	D	Inhibition of Aβ oligomer formation *in vitro*, effective in AD tg mice after oral application	Frydman-Marom *et al*., 2009 [103]
none	** EIVY**-rest	β-sheet breaker see text in subchapter “*KLVFF derived β-sheet breakers*”	L	Modulate Aβ aggregation, depending of solvent disruptive amino acid sequence. Discussed in this article with KLVFF-based β-sheet breakers	Sun *et al*., 2009 [76]

D/L: describes peptide conformation. AIB: α-aminoisobutyric acid; tg: transgenic. Aβ-derived peptide sequences are written in bold.

**Table 2F.** Peptides Influencing Aβ Aggregation and Toxicity. Peptides Selected Using Combinatorial Libraries

Name	Sequence	Description	D/L	Results	Reference

DP1 – DP8	6 residues from: Ala, Ile, Val, Ser, Thr, Gly	“Decoy” peptides of combinatorial library	D	Eliminate calcium effect of Aβ1-42	Blanchard *et al*., 97, 2000 [106, 107]

None	Several with 4 groups of consensus sequences:	Out of combinatorial library	L	Inhibit Aβ aggregation	Schwarzman *et al*., 2005 [108]
	++H+(H/+)				
	or				
	++XX+				
	or				
	(D/E)LVH				
	or				
	+LVLF				

None	12 mers	Phage Display with two libraries.	L	Ligands bind targets in different aggregation states, partly affect aggregation	Orner *et al*., 2006 [109]
		Parent molecule: KLVFFKKKKKK			
		Target: Monomeric and fibrillar Aβ.			

ANA1	6 or 15 mers	Novel selection involving phage display and counterselection against rat Aβ	L	Inhibit Aβ toxicity	Taddei *et al*., 2008 [110]
ANA2	e.g. TNPNRRNRTPQMLKR (ANA1)				
ANA3					

1A, 1B, 2	MSNKGASIGLMAGDVDIADSHA or	Combinatorial library and selection based on enzyme EGFP	L	Inhibitory effect on Aβ aggregation, de-fibrillization	Bain *et al*., 2009 [111]
	MSNKGASNALMAGDGDIADSHS or				
	MQKLDVVAEDAGSNK				

D3	RPRTRLHTHRNR	Mirror image phage display	D	Modulates Aβ oligomerization, effective in tg mouse model after oral application	Van Groen *et al*., 2008; Funke *et al*., 2010 [112, 113]

JM169	Trimer-TEG-D3	Hybrid compound D3 and nonpeptidic β-sheet breaker	D	*In vitro* more effective than trimer or D3	Müller-Schiffmann *et al*., 2010 [115]

1-8	e.g. RAPMGGR or	Panning of heptapeptide library against soluble Aβ1-42	L	Suppress Aβ1-42 37/48 kDa oligomer formation, keep monomer stable	Kawasaki *et al*., 2010 [128]
	RRPVVGR				

GN-peptide	GNLLTLD	Peptide homologous to sequence from apo A-I, selected by phage display	L	Modulates aggregation, prevents hippocampal neuronal cultures from Aβ induced degeneration	Paula-Lima *et al*., 2009 [119]

D-4F	Ac-DWFKAFYDKVAEKFKEAF-NH_2_	Apo A-I mimetic peptide	D	Inhibits Aβ deposition and improver cognitive performance of AD tg mice after oral application	[120]

D/L: describes peptide conformation. TEG: triethyleneglycol. +: positively charged amino acids. H: hydrophobic amino acid. X: hydrophobic or polar amino acid
